# Drug-Induced Sleep Endoscopy for Targeted Sleep Surgery in Pediatric Patients

**DOI:** 10.7759/cureus.84122

**Published:** 2025-05-14

**Authors:** Theresa A Schneider, Anya Costeloe, Ani Mnatsakanian, Jacob Surma, Suzanne Forman, Michael Haupert, Prasad John Thottam

**Affiliations:** 1 Otolaryngology, Ascension Health, Warren, USA; 2 Otolaryngology, Corewell Health, Royal Oak, USA; 3 Otolaryngology, Central Michigan University College of Medicine, Mount Pleasant, USA

**Keywords:** dise, pediatric tonsillectomy and adenoidectomy, sleep disordered breathing, supraglottoplasty, tonsillar hypertrophy

## Abstract

Introduction: This study aims to evaluate the efficacy of drug-induced sleep endoscopy (DISE)-targeted surgery to identify the locations of obstruction and to determine how DISE findings influence whether the standard of care surgery, adenotonsillectomy, is performed.

Methods: This prospective cohort study was done at an academic children's hospital. All patients (n = 42) underwent polysomnography. DISE was performed to evaluate tonsil and adenoid size, Yellon tongue base, lateral pharyngeal wall (LPW) collapse, and signs of laryngomalacia. Surgery was performed based on the most prominent locations of obstruction. Pre-operative and post-operative University of Michigan Pediatric Sleep Questionnaire (UMPSQ) was given to determine the likelihood of residual OSA.

Results: Surgeries included tonsillectomy, adenoidectomy, lingual tonsillectomy, laryngeal cleft repair, supraglottoplasty, and turbinate reduction. Patients had improvement in the UMPSQ score from 13.36 ± 3.67 to 5.68 ± 3.46 (P=0.05). Those who underwent adenotonsillectomy had a greater decrease in UMPSQ scores than those who did not (P=0.03). Patients with significant LPW collapse were more likely to have adenotonsillectomy (P=0.001), while patients with higher Yellon tongue base scores were less likely (P=0.005). There was no statistically significant relationship between OSA severity and whether adenotonsillectomy was performed.

Conclusions: DISE is a valuable tool for evaluating children with multi-level obstruction, and findings change surgical decision-making for children without enlarged tonsils. Adenotonsillectomy resulted in the greatest decrease in OSA symptoms, but was mainly performed on patients with significant LPW collapse.

## Introduction

Obstructive sleep apnea (OSA) and sleep-disordered breathing (SDB) are prevalent in 2-5% and up to 35% of the general pediatric population, respectively [1]. Untreated OSA can lead to detrimental effects in children, including daytime sleepiness, poor cognition, cardiovascular derangements, neurobehavioral issues, and even growth abnormalities [2-4]. While continuous positive airway pressure (CPAP) is considered the gold standard for treating OSA, compliance in pediatric populations is generally poor [4,5]. Thus, surgical intervention targeting anatomic obstruction commonly manages pediatric patients with OSA and SDB. Current recommendations suggest the removal of tonsils and adenoids as the first-line treatment of moderate-severe pediatric OSA [6,7]. The current guidelines indicate drug-induced sleep endoscopy (DISE) as a secondary measure, should OSA or OSA symptoms persist after adenotonsillectomy [8].

Adenotonsillectomy has been shown to effectively improve the apnea-hypopnea index (AHI) and sleep-related quality of life [9]; however, not all patients with symptoms of SDB or positive sleep studies present with classical physical exam findings of adenotonsillar hypertrophy. Often, these pediatric patients are more clinically challenging. Drug-induced sleep endoscopy has become an increasingly popular technique to evaluate the airway and the level of obstruction in children with clinically non-enlarged tonsils and adenoids [10]. Past studies using DISE have demonstrated that non-enlarged tonsils (size 1+ to 2+) may not cause obstruction [11] and have questioned adenotonsillectomy as the first-line treatment. Since its introduction in 1991 [12], DISE has been an effective tool in optimizing surgical intervention for pediatric OSA. A recent study by He et al. evaluated pre-operative and post-operative AHI in children with a history of adenotonsillectomy undergoing DISE-directed surgery and found a significant decrease in AHI following surgery [13]. Other studies have noted its usefulness in surgically naïve patients, where 58% of patients with OSA underwent treatment that did not include adenotonsillectomy due to DISE findings [14].

This study aims to determine the anatomical level of obstruction in children undergoing DISE. A secondary aim is to determine whether there is a correlation between the location of obstruction and the surgery performed on clinical outcomes. An additional point of interest is whether utilizing DISE as an initial treatment tool will affect the types of surgeries performed for pediatric OSA.

This article was previously presented as a meeting abstract at the AAO-HNSF Annual Meeting & Oto Experience on October 6, 2020; the MSU COM Research Day on April 11, 2024; and the CMED Research Symposium on April 26, 2024.

## Materials and methods

Study design and participants

This prospective cohort study was performed on patients who underwent DISE from January 2017 to October 2017 at a tertiary care pediatric hospital. All study protocols were approved by the study hospital's Institutional Review Board (IRB). Subjects completed the validated pre-operative University of Michigan Pediatric Sleep Questionnaire (UMPSQ) [15]. Inclusion criteria were patients aged six months to 18 years diagnosed with OSA or sleep-disordered breathing. Exclusion criteria were pregnancy and patients with Grade 4 tonsils. These patients were assumed to have OSA or SDB due to obvious tonsillar hypertrophy. All children were scheduled for DISE before surgical intervention for sleep-disordered breathing and OSA. OSA severity was defined based on AHI per practice guidelines: mild was one to less than five events per hour, moderate was five to less than 10 events per hour, and severe was greater than 10 events per hour.

Validated pediatric sleep questionnaire

The pre-operative and post-operative UMPSQ was given to determine the likelihood of residual OSA. Before surgery, parents or guardians of subject participants completed a 22-question validated pediatric sleep questionnaire. The total number of yes responses was calculated to give a pre-operative score. Parents were asked to complete the survey again at least four weeks post-operatively. Post-operative surveys were either filled out at the post-operative office visit or were completed via telephone.

DISE-directed surgery

**Figure 1:**
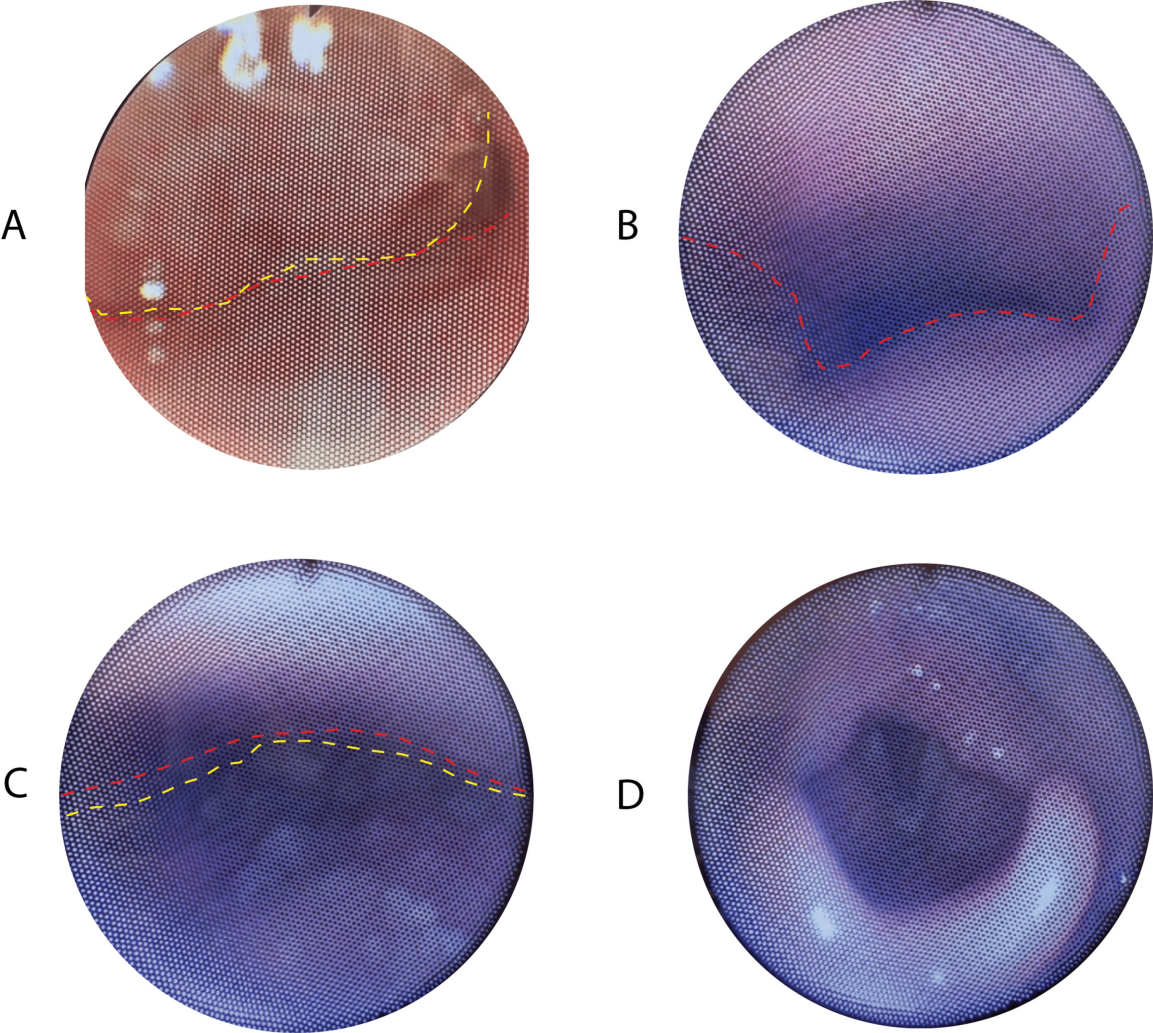
Endoscopic views obtained during drug-induced sleep endoscopy demonstrating multiple levels of upper airway assessment (patient underwent adenoidectomy and lingual tonsillectomy). (A) Nasopharyngeal view showing significant adenoid hypertrophy (outlined in yellow) with obstruction at the junction of the posterior nasal cavity floor (demarcated in red), resulting in narrowing of the nasopharyngeal airway. (B) Oropharyngeal view demonstrating patent lateral walls with the absence of palatine tonsillar obstruction. Anterior pharyngeal wall contour outlined in red shows maintenance of airway patency at this level. (C) Inferior oropharyngeal view revealing complete collapse of the base of tongue due to lingual tonsillar hypertrophy (tongue base outlined in yellow), causing significant airway obstruction against the posterior pharyngeal wall (marked in red). (D) Supraglottic view demonstrating laryngeal structures with mild prolapse of the arytenoid cartilage and surrounding supraglottic structures.

Drug-induced sleep endoscopy was performed at the children's hospital or the outpatient surgery center. DISE technique was performed as described by Wooten et al. [16]. Once in the operative suite, patients are masked under sevoflurane/nitrous oxide inhalation anesthetic. Intravenous access is then achieved, and propofol is delivered in 1 mg/kg boluses by the anesthesia department to achieve a level of consciousness that mimics natural sleep (asleep but spontaneously breathing). A flexible fiberoptic endoscope was passed through the nose, and the airway was assessed (Figure 1). Findings recorded during DISE included tonsil size, adenoid size, Yellon tongue base score [17], lateral pharyngeal wall (LPW) collapse, and evidence of laryngomalacia. Adenoids were graded in percentage obstruction (0-100%). Tonsils were graded on the percentage of obstruction (0-100%). LPW collapse was determined to be present or absent. The Yellon tongue base score was calculated, as described by Yellon; Grade 0 is a normal airway, Grade 1 is prolapse of the epiglottis along the posterior pharyngeal wall with normal positioning of the tongue, Grade 2 is prolapse of the epiglottis and base of the tongue with only epiglottic tip visible, and Grade 3 is glossoptosis with no portion of the epiglottis visible [17]. Laryngomalacia was assessed as present or absent after viewing the supraglottis in each patient. Surgery was performed per the surgeon's clinical judgment based on the location of the obstruction. There were two separate surgeons whose data were included in this study. Surgeries included tonsillectomy, adenoidectomy, lingual tonsillectomy, lateral pharyngoplasty, and supraglottoplasty.

Statistical analysis

Patient charts were reviewed for demographics, including age and sex, pre-operative AHI, and pre- and post-operative sleep quality of life scores. Findings on DISE, as described above, and the surgical procedures performed were recorded. Statistical analysis was performed using the SAS System for Windows version 9.4. Frequency and percentages were determined for DISE findings, including tonsil size, adenoid size, Yellon tongue base score, laryngomalacia, and LPW collapse. LPW collapse present was compared to tonsil size, and percentages of collapse present were determined for 1+, 2+, and 3+ tonsils. Patients with 4+ tonsils were omitted because these patients did not need DISE and typically underwent tonsillectomy.

For variables with a normal distribution, mean and standard deviation were reported, and group comparisons used a two-sample t-test and a 95% confidence interval for the difference in means. The median was calculated for continuous variables without a normal distribution, and p-values for group comparisons were obtained using a Wilcoxon Rank-Sum test. A comparison of whether the surgery performed was the recommended adenotonsillectomy with LPW, AHI, and tonsil size was done using an exact version of Pearson's chi-square test and an exact 95% confidence interval for the odds ratio.

## Results

There were 42 patients who met the inclusion criteria. Of the study participants, 10 were females (23.8%) and 32 were males (76.2%). Ages ranged from 1-17 years, and the median age was seven. Thirty-two patients had pre-operative polysomnograms (PSGs), and of these patients, 16 had mild OSA (38.1%), 12 had moderate (28.6%), and 14 had severe OSA (33.3%). The median AHI was 6.8 (range: 1-106) (Table 1).

**Table 1:** Patient demographics and OSA severity. Note: AHI = Apnea-Hypopnea Index, OSA = Obstructive Sleep Apnea Percentages represent the percentage of patients in the indicated group.

Category	Sub-category	Value
Age (years)	Median	7
	Range	1-17
AHI	Median	6.8
	Range	1-106
Gender	Female	10 (23.8%)
	Male	32 (76.2%)
OSA Severity	Mild	16 (38.1%)
	Moderate	12 (28.6%)
	Severe	14 (33.3%)

Pre-operative DISE findings were recorded, and nine patients (21.4%) had 1+ tonsils, 24 patients (57.1%) had 2+ tonsils, and nine patients (21.4%) had 3+ tonsils. The Yellon tongue base score was 0 in 11 patients (26.2%), one in 11 patients (26.2%), two in 12 patients (28.6%), and three in eight patients (19.1%). Laryngomalacia was identified on endoscopy in seven patients (16.7%). LPW collapse was noted in 21 patients (50%) (Table 2). Surgeries were performed based on anatomic obstruction shown with DISE and are summarized in Table 3.

**Table 2:** Pre-operative drug-induced sleep endoscopy (DISE) findings. Note: N = Number of patients, LPW = Lateral Pharyngeal Wall Percentages represent the percentage of patients in the indicated group.

Finding	Sub-category	Value
Adenoid Obstruction	yes	29 (31.0%)
	no	13 (69.1%)
Tonsil Size	1+	9 (21.4%)
	2+	24 (57.1%)
	3+	9 (21.4%)
Yellon Tongue Base	0	11 (26.2%)
	1	11 (26.2%)
	2	12 (28.6%)
	3	8 (19.1%)
LPW Collapse	Yes	21 (50%)
	No	21 (50%)
Laryngomalacia	Yes	7 (16.7%)
	No	35 (83.3%)

**Table 3:** Surgeries performed. Note: N = Number of Patients, % = Percentage of patients treated with the procedure

Surgery Performed	N	%
Tonsillectomy	23	54.76%
Adenoidectomy	35	83.33%
Lingual tonsillectomy	16	38.10%
Supraglottoplasty	5	11.90%
Laryngeal cleft	2	4.76%
Turbinate reduction	2	4.76%

LPW collapse was associated with increasing tonsil size. Of patients with 1+ tonsils, 22.2% had LPW collapse, 41.7% of patients with 2+ tonsils had LPW collapse, and 100% of patients with 3+ tonsils had LPW collapse. A statistically significant positive correlation existed between tonsil size and LPW collapse (P=0.0015). There was a slightly inverse correlation between tonsil size and OSA severity. Patients with smaller tonsils were more likely to have severe OSA; 66.7% of 1+ tonsils had severe OSA, while only 22.2% of patients with 3+ tonsils had severe OSA (Table 4). Over half of the patients did not have the academy guidelines-recommended adenotonsillectomy (T&A) (n=27, 64.3%). Patients with larger tonsils were more likely to have adenotonsillectomy; 89% of patients with 3+ tonsils had a T&A, 25% with 2+ tonsils had a T&A, and only 11% with 1+ tonsils had T&A (P=0.001). However, patients with higher Yellon tongue base scores were less likely to have T&A; 12.5% of patients with a Yellon tongue base score of 3 had a T&A compared to 64% of patients with Yellon 0 (P=0.005). There was insufficient information to conclude the existence of a relationship between OSA severity and whether adenotonsillectomy was performed (P=0.42) (Table 4).

**Table 4:** Relationship of tonsil size with LPW collapse and OSA severity. Note: N = Number of patients, % = Percentage of patients within each tonsil size category exhibiting the specified condition, LPW = Lateral Pharyngeal Wall, OSA = Obstructive Sleep Apnea

Tonsil Size	N	LPW Collapse (N, %)	Mild OSA (N, %)	Moderate OSA (N, %)	Severe OSA (N, %)
1+	9	2 (22.2%)	2 (22.2%)	1 (11.1%)	6 (66.7%)
2+	24	10 (41.7%)	10 (41.7%)	8 (33.3%)	6 (25%)
3+	9	9 (100%)	4 (44.4%)	3 (33.3%)	2 (22.2%)

UMPSQ findings

Twenty-five patients (60%) completed the post-operative survey. The remainder of the patients could not be reached despite continuous efforts. Overall, patients had improved in UMPSQ scores from 13.4 ± 3.7 prior to surgery to 5.7 ± 3.5 after surgery. The average change from pre-operative score to post-operative score was 7.7 ± 4.5. Patients who underwent adenotonsillectomy had a more significant decrease in scores than those who did not; the average change for patients who had adenotonsillectomy was 9.8 ± 4.5, while the average change for patients who did not was 6 ± 3.9 (P = 0.032). There was no clear relationship between tonsil size and the amount of improvement in survey results. Similarly, there was no well-defined relationship between pre-operative LPW collapse or OSA severity and change in UMPSQ scores (Table 5). Patients who filled out the post-op survey had slightly higher average pre-operative survey scores (13.4 vs. 11.9) and were more likely to have tonsillectomy (72% of patients who only underwent tonsillectomy completed a post-op survey, while 43% of patients who had other surgeries completed the post-operative survey). Patients who had severe OSA were also more likely to complete the post-op survey (completion: 79% for severe, 50% for mild and moderate). Patients with LPW were more likely to complete the post-op survey, 71% with LPW versus 48% without LPW collapse. Post-op surveys were available for 44%, 58%, and 78% of patients with 1+, 2+, and 3+ tonsils, respectively.

**Table 5:** Mean change from pre-operative to post-operative UMPSQ surveys based on surgery performed, OSA severity, tonsil size, and LPW status. Note: T&A = Tonsillectomy and Adenoidectomy, OSA = Obstructive Sleep Apnea, SD = Standard Deviation, LPW = Lateral Pharyngeal Wall

Group	N	Change in survey score	SD
All patients	25	7.68	4.99
Surgery performed			
Not T&A	14	6.0	3.88
T&A	11	9.82	4.45
P-value			0.032
OSA severity			
Mild	8	10	4.5
Moderate	6	6.33	3.08
Severe	11	6.73	4.80
P-value			0.21
Tonsil size			
1+	4	7.75	5.19
2+	14	7.29	4.89
3+	7	8.43	3.78
P-value			0.87
LPW collapse			
No	10	6.20	3.88
Yes	15	8.67	4.72
P-value			0.18

## Discussion

This study examines the application of DISE in treating pediatric OSA or SDB. The primary objective is to identify the anatomical site of obstruction and explore its correlation with OSA severity and treatment outcomes. The effectiveness of the surgical intervention was evaluated through validated sleep surveys administered before and after the procedure. Despite 60% compliance in completing the post-operative PSQ, the results demonstrated that surgery successfully addresses OSA and sleep-disordered breathing, improving the quality of life. The average post-operative score was significantly less than the pre-operative score (mean decrease of 7.7 ±4.5; P=0.05). Prior studies have similarly demonstrated efficacy in DISE-directed surgery, as measured by a significant decrease in AHI [13].

Most patients who underwent DISE had clinically non-enlarged tonsils; 79.6% had 1+ or 2+ tonsils. This is valuable to note as children with a history consistent with OSA and/or a positive sleep study often have large adenoids that may not be visualized on a physical exam unless a fiberoptic scope is performed. However, clinically non-enlarged tonsils should not be, by default, considered non-obstructive. LPW collapse was noted in 22.2% of patients with 1+ tonsils and 41.7% with 2+ tonsils, which are usually considered non-enlarged on physical examination. This serves as important clinical information for all pediatric providers in the work-up and management of OSA - those with non-enlarged tonsils may still require and benefit from surgical intervention. Various surgical procedures were done based on intraoperative DISE findings. Therefore, surgery was categorized as recommended versus not recommended based on the current academy guidelines for treating pediatric OSA. While recommended surgery of adenotonsillectomy was often performed, 64.3% of children had findings on DISE that necessitated more complex surgery than adenotonsillectomy, such as supraglottoplasty, lingual tonsillectomy, laryngeal cleft surgery, turbinate reduction, or a combination of these procedures. Those with larger tonsils were more likely to have a T&A performed, and those with a higher Yellon tongue base score were less likely to have a T&A performed. Additionally, there was insufficient information to conclude the existence of a relationship between OSA severity and whether T&A was performed. According to a 2017 study by Gazzaz et al., DISE findings resulted in a deviation from the recommended adenotonsillectomy in 35% of patients [18].

Similarly, a 2020 study by Kirkham et al. reported a deviation rate of 58% from the standard of care, with both studies classifying adenoidectomy alone or tonsillectomy alone as a deviation [14]. This aligns with the findings of the current study. This study adds to the current literature as additional surgical interventions were performed based on DISE findings, other than just tonsillectomy, adenoidectomy, or adenotonsillectomy. Lingual tonsillectomy was the most common intervention performed outside of adenotonsillectomy (38.1%).

When comparing whether the recommended surgery was done for varying levels of OSA severity, the data revealed that AHI may be lower in patients undergoing adenotonsillectomy. However, this was not statistically significant (P=0.42). As expected, larger tonsil size did correlate with an increased likelihood of adenotonsillectomy. Meanwhile, higher Yellon tongue base scores correlated with less likelihood of having the recommended adenotonsillectomy (P=0.005). This corroborates previous research suggesting that children with non-enlarged tonsils will likely have an obstruction at other sites [11,19].

Interestingly, patients who underwent the recommended adenotonsillectomy had significantly higher changes from pre-operative to post-operative PSQ scores than those who underwent other surgeries. This may indicate that obstruction at the level of the adenoids and tonsils is more accessible to treat than obstruction further down in the airway. However, more patients who had adenotonsillectomy completed the post-operative survey; 72% of those who underwent adenotonsillectomy, versus only 43% of patients who had other surgeries, completed the post-operative survey. Patients who had severe OSA were also more likely to complete the post-operative survey (completion: 80% for severe, 50% for mild and moderate). Further research with a larger sample size is warranted to address this.

This study has several limitations that need consideration. First, not all patients underwent pre-operative polysomnography (PSG), and post-operative PSGs were not conducted. The reason pre-operative PSG may not have been conducted was due to positive symptoms of OSA reported by the parent or guardian. This included symptoms, such as the presence of apneas, snoring, daytime sleepiness, morning headache, and restless sleeping. Since the utility of UMPSQ scores relies on patient compliance, obtaining pre-operative and post-operative PSG data would enable a more comprehensive analysis beyond subjective scoring. Only 25 out of 42 patients completed the post-operative UMPSQ, and a larger sample size might reveal more statistically significant correlations.

Furthermore, the absence of a control group is another limitation. Future research could benefit from including a control group undergoing standard-of-care adenotonsillectomy for comparative purposes. This would provide a valuable benchmark for evaluating the outcomes of the DISE-guided surgical interventions. Additionally, BMI should be included in the data statistics, as it may result in poorer outcomes in patients with higher BMI scores.

## Conclusions

The findings of this study underscore the significance of DISE as a valuable tool in assessing multi-level obstruction in pediatric patients experiencing OSA or sleep-disordered breathing. The study highlights the ongoing relevance of the UMPSQ as a valuable resource for evaluating OSA symptoms and residual OSA following surgical intervention.

Consistent with earlier research, the study emphasizes that DISE frequently leads to a modification in surgical management, shifting from the traditional approach of adenotonsillectomy to addressing other levels of anatomical obstruction. This holds particular significance for patients with non-enlarged tonsils and adenoids experiencing obstruction at a different subsite of the airway. This study aims to demonstrate the important utilization of DISE as an initial treatment tool in pediatric patients undergoing surgical evaluation and treatment for pediatric OSA.
